# Visual outcomes and choroidal thickness associated with human leukocyte antigen DRB1*04 in unclassifiable uveitis in Japanese patients

**DOI:** 10.1186/s12886-021-02222-9

**Published:** 2021-12-28

**Authors:** Norihiko Misawa, Mizuki Tagami, Atsushi Sakai, Takeya Kohno, Shigeru Honda

**Affiliations:** Department of Ophthalmology and Visual Sciences, Graduate School of Medicine, Osaka City University, 1-5-7 Asahimachi, Abeno-ku, Osaka-shi, Osaka, 545-8586 Japan

**Keywords:** Non-infectious uveitis, Unclassifiable uveitis, Human leukocyte antigen, HLA-DRB1*04, Asian patients, Visual acuity

## Abstract

**Purpose:**

Human leukocyte antigen (HLA) and immunity are related. Uveitis is also closely related to immunity. For example, the common presence of human leukocyte antigen (HLA)-DRB1*04 in the immune response is well known. The aim of this study was to investigate the relationship between visual prognosis and various HLA alleles before and after therapy in patients with unclassifiable uveitis, excluding those with Vogt-Koyanagi-Harada (VKH) disease.

**Methods:**

This retrospective case series included 42 eyes from 22 consecutive patients with unclassifiable uveitis, excluding those with VKH disease. Visual acuity (VA), sex, refractive error, central retinal thickness (CRT), central choroidal thickness (CCT), and duration from onset to treatment were measured at initial and 6-month visits. Mean values of parameters were compared at each visit. Genotyping was performed by polymerase chain reaction amplification with sequence-specific primers.

**Results:**

DRB1*04 showed a dominant change. No significant difference was observed in the other alleles. In DRB1*04, The mean differences in initial CCT, 6-month CCT, and 6-month VA showed statistically significant difference was found in best-corrected visual acuity (BCVA) between DRB1*04+ and DRB1*04− at the first visit. BCVA values at baseline and at the final visit were 0.13 ± 0.29 and 0.20 ± 0.36 in the DRB1*04+ and 0.00045 ± 0.20 and − 0.058 ± 0.11 in the DRB1*04− groups(*p* = 0.00465). Central Choroidal Thickness (CCT) values pretreatment and at the final visit after treatment were (pretreatment:361.00 ± 361.0 μm,after treatment: 286.00 ± 106.53 μm, *p* = 0.0174) in the DRB1*04+ group, and (pretreatment:281.3 ± 139.68 μm,after treatment:223.85 ± 99.034 μm, *p* = 0.0426) in the DRB1*04− group, respectively, indicating changes between baseline and the final visit. CCT was significantly greater in the DRB1*04+ group at both the initial visit and at 6 months. Multivariate analysis showed a significant difference between the presence or absence of DRB1*04 and sex.

**Conclusion:**

HLA-DRB1*04 allele may affect visual prognosis and CCT in unclassifiable uveitis.

**Supplementary Information:**

The online version contains supplementary material available at 10.1186/s12886-021-02222-9.

## Introduction

Uveitis is an inflammatory condition of the iris, ciliary body, choroid, and surrounding tissues [1]. Causes can be categorized broadly as infectious or non-infectious. The most common cause of uveitis in Japan is sarcoidosis, followed by Vogt-Koyanagi-Harada (VKH) disease. Other common conditions include Behçet’s disease, acute anterior uveitis, scleritis, and herpetic iritis [2–4].

Inflammation of the uvea has many causes including autoimmunity and infection [5–7]. In particular, the pathogenesis of uveitis involves an immune system imbalance and the genetic background [8–10]. In general, some kind of systemic immune-disease including Rheumatic disease were associated with Human leukocyte antigen (HLA) [11, 12]. HLA is also the most common genetic background for several types of uveitis [8, 13].

The HLA system is a locus of genes encoding the major histocompatibility complex (MHC) and represents a set of cell surface molecules that mediate leukocyte interactions [13].

The uvea is rich in blood flow and a place where leukocytes and proteins such as antigens can easily accumulate and cause an inflammatory response in several types of uveitis associated with autoimmunity [14–16]. Therefore, in uveitis, HLA is very important for disease identification and disease progression, and is being studied and attracting the attention of clinical researchers.

A link to HLAB27 and its pathogenesis has been reported in ankylosing spondylitis [17]. Behçet’s disease and VKH disease are associated with HLA [18]. In addition, we previously reported that HLA alleles may confer a different visual prognosis in VKH disease [19]. However, no previous reports have been published about unclassified intraocular inflammation and a possible association with HLA and visual functions.

We are interested in the possibility that HLA is associated with visual function even in unclassifiable uveitis. In this study, we investigated whether changes in visual functions and ocular structures are associated with various HLA in unclassifiable uveitis, excluding VKH disease, in east Asian patients.

## Methods

This retrospective case series included 42 eyes from 22 consecutive patients with uveitis, excluding those with VKH disease, who visited the Ophthalmology Department at Osaka City University Hospital between December 2009 and January 2020 and were followed up for more than 6 months after the start of systemic corticosteroid therapy. The images were followed up in the outpatient clinic at different intervals until the visit. The other eye was not followed up with images and was removed in two patients (No. 15.20). The study was performed according to the tenets of the Declaration of Helsinki. Approval for this study was obtained prior to the start of the study from the institutional review board at Osaka City University, Japan. Written informed consent for the storage of patient information in the hospital database and use in research was provided by all patients enrolled in the study. All patients underwent a comprehensive ophthalmologic examination, which included measurement of BCVA using a Landolt C acuity chart at 5 m, indirect ophthalmoscopy, slit-lamp biomicroscopy, fundus camera examination (Topcon, Tokyo, Japan), and optical coherence tomography (OCT) (spectral-domain OCT, Spectralis; Heidelberg Engineering, Heidelberg, Germany). Horizontal and vertical scans crossing the fovea and volume scans in the macular were performed. Enhanced depth imaging protocols were used on Spectralis OCT. VKH disease was diagnosed according to the criteria of Sugiura and the VKH Disease Committee [20]. Patients with non-ocular symptoms, such as headaches, were excluded. All 22 patients had no depigmentation findings even after 6 months. None of the patients had any medical or ocular history at the initial visit. Changes in central retinal thickness (CRT) and central choroidal thickness (CCT) were assessed by vertically and horizontally oriented enhanced depth imaging optical coherence tomography (OCT) of the macula (Spectralis HRA + OCT Heidelberg Engineering, Heidelberg, Germany) for up to 3 months after treatment. The CRT was defined as the distance between the inner surface of the retina and the inner boundary of the retinal pigment epithelium (RPE). The CCT was measured from the hyper-reflective line corresponding to the RPE to the outer border of choroid. CRT and CCT were measured manually with a built-in scale using OCT images through the central fovea. Measurements of CRT and CCT in each eye were then reconfirmed by three experts (NM, AS, and MT) by checking the OCT images. They were categorized by those with and without a specific allele. For example, the group with HLA -DRB1*04 was designated (DRB1*04+), and the group without HLA- DRB1*04 was designated (DRB1*04−), and the two groups were compared. HLA antigens were extracted from blood samples. Blood samples were taken by a specialist technician in an outpatient clinic. HLA antigens were identified by a commercially available Micro-SSP (sequence specific primers) kit (One Lambda Inc.、CA). Specific Primers sequences described previous at home page (https://www.thermofisher.com/onelambda/wo/en/products.html?articleNumber=SSPABDR). DNA was extracted from the collected blood. D-mix and AmpliTaq DNA Polymerase (ThermoFisher, CA) were added to the DNA. The solution was dispensed into a defrosted typing tray, and PCR was performed. Electrophoresis was performed on an 2.5% agarose gel, and the results were determined using software (HLA Fusion,One Lambda Inc.、CA). In HLA typing by polymerase chain reaction amplification with sequence-specific primers (PCR-SSP), typing specificity is part of the amplification step. Exclusion criteria were as follows: 1) history of traumatic ocular injury; 2) history of intraocular surgery; 3) presence of high myopia > − 9.0 D; 4) diagnosis of classifiable uveitis including VKH, Behchet’s disease, Ocular Sarcoidosis [20–22]. 5) glaucoma involving ocular hypertension.

Mann-Whitney U test and Fisher’s exact test were used to analyze and compare baseline patient characteristics. Multivariate analysis was performed on Fist CCT, First CRT, First CCT, First BCVA, gender and age.

Statistical analyses were performed using SPSS Statistics version 22 software (IBM Japan, Tokyo, Japan). Values of *p* < 0.05 were considered statistically significant.

## Results

### Patient characteristics in HLA-DRB1*04

Of the 22 people with unclassifiable uveitis in this study, 10 men and 12 women were enrolled. Two eyes were excluded because they had not been examined. The mean age was 55.63 ± 17.57 years in the DRB1*04+ group and 51.50 ± 20.35 years in the DRB1*04− group. The other parameters are summarized in Table 1. Treatment options (steroid eye drops, sub-Tenon’s triamcinolone acetonide injection, and oral steroids) and HLA-DRB1* genotyping results are summarized in Table 2. Eleven patients (50%) were HLA-DRB1*04+ (homozygous: *n* = 1; heterozygous: *n* = 10; Table 2).

**Table 1:** Baseline patient characteristics in HLA-DRB*04

Participant baseline characteristics	HLA-DRB1*04(+)	HLA-DRB1*04(−)	*P* value
Males	6	4	0.67^※1^
Females	5	7	
Age (years)	52.63 ± 17.57	51.50 ± 20.35	0.93^※2^
BCVA (logMAR)	0.12 ± 0.29	0.00045 ± 0.20	0.102^※2^
CRT (μm)	351.55 ± 270.66	310.5500 ± 143.2368	0.65^※2^
CCT (μm)	**361.0 ± 361.0**	281.3 ± 139.68	0.0174^*※2^

※1 Fisher’s exact test (SEX)

※2 Mann–Whitney U test (Age, BCVA, CRT, CCT)

*P<0.05

**Table 2:** Clinical parameters

ID	Sex	Age (years)	Classification	HLA Allele			
				HLA-A	HLA-B	HLA-DR	HLA-DQ
1	M	47	Posterior uveitis	24:31	54:56	**4:9**	4:3
2	F	79	Panuveitis	24:24	61:61	8:9	6:3
3	M	72	Panuveitis	2:26	46:48	**4:9**	4:9
4	F	67	Posterior uveitis	2:24	61:46	15:9	6:9
5	M	81	Panuveitis	24:24	7:52	15:4	6:4
6	F	49	Posterior uveitis	24:26	62:54	12:14	7:9
7	F	47	Posterior uveitis	26:31	62:35	8:14	6:5
8	F	71	Panuveitis	24:26	61:46	**4:9**	8:9
9	M	36	Panuveitis	24:24	61:46	**4:12**	8:9
10	F	70	Panuveitis	2:24	46:60	**4:8**	8:4
11	M	47	Anterior Uveitis	2:24	51:51	**4:9**	4:9
12	M	62	Panuveitis	2:2	13:61	9:12	7:9
13	F	38	Posterior uveitis	2:24	51:61	**4:15**	7:9
14	M	76	Panuveitis	26:26	61:61	9:9	9:9
15	M	47	Anterior Uveitis	24:24	27:52	1:9	5:9
16	F	63	Posterior uveitis	24:26	39:61	15:9	6:9
17	F	33	Posterior uveitis	1:2	37:39	15:10	6:5
18	M	43	Anterior Uveitis	2:24	61:52	**4:15**	4:6
19	F	49	Posterior uveitis	24:26	54:59	**4:4**	4:4
20	M	26	Panuveitis	24:24	35:61	8:14	8:5
21	F	45	Intermediate uveitis	24:24	7:52	1:15	5:6
22	F	25	Panuveitis	2:26	35:55	**4:14**	4:5

The list of the Alleles of HLA-A, −B and -DR, DQ. We identified 5 patients as HLA- A * 24:02 the most common of the HLA- A .11 patients had DRB1*04,8 patients had A * 26,DRB*9 had 10 patients and only 2 people as B*51

### Visual outcomes and anatomical outcomes with OCT in HLA-DRB1*04

**Fig. 1:**
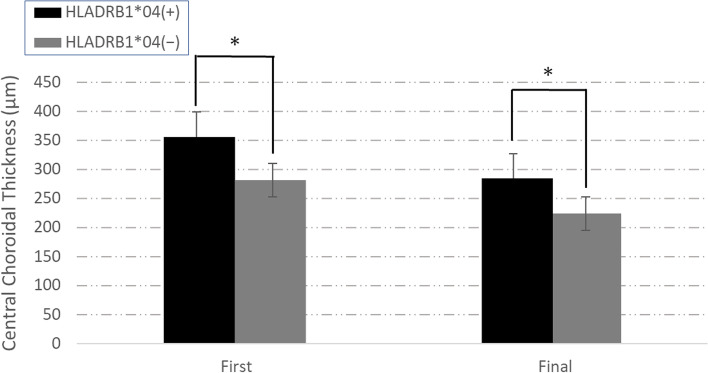
Visual acuity (BCVA, LogMAR) at the first visit (First) and the 6-month visit (Final). No significant difference was found between HLA-DRB1*04+ and HLA-DRB1*04− at the first visit; the HLA-DRB1*04+ group showed a worsening trend from the first visit. At the final visit, the HLA-DRB1*04− group showed a superior improvement in visual acuity compared to the HLA-DRB1*04+ group. *P<0.05

For visual acuity, mean log MAR best-corrected visual acuity (BCVA) values at baseline and at the final visit were 0.13 ± 0.29 and 0.20 ± 0.36 in the DRB1*04+ and 0.00045 ± 0.20 and − 0.058 ± 0.11 in the DRB1*04− groups, respectively. A statistically significant difference in final visual acuity was identified (*p* = 0.00465) (Fig. 1). No significant difference in BCVA was found between the DRB1*04+ and DRB1*04− groups at the initial diagnosis; however, a significant difference was present after treatment.

**Fig. 2:**
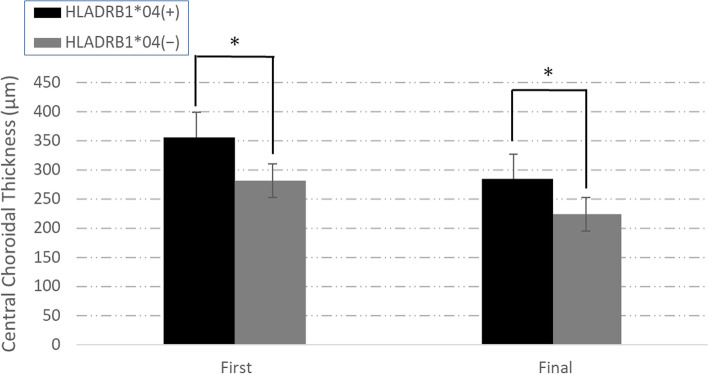
The CCT was thicker in the HLA-DRB1*04+ group than the HLA-DRB1*04− group at the first and the final visits. Both the HLA-DRB1*04+ and HLA-DRB1*04− groups showed a decrease in CCT after treatment. *P<0.05

In anatomical measurements with OCT, mean CCT values pretreatment and at the final visit after treatment were (pretreatment:361.00 ± 361.0 μm, aftertreatment:286.00 ± 106.53 μm, *p* = 0.0174) in the DRB1*04+ group, and (pretreatment:281.3 ± 139.68 μm, aftertreatment: 223.85 ± 99.034 μm, *p* = 0.0426) in the DRB1*04− group, respectively, indicating changes between baseline and the final visit. Mean post-treatment CCT values were significantly decreased compared with baseline CCT (Fig. 2). In CCT change, there were no difference between 2 groups (DRB1*04+ group:72.86 ± 116.87 μm, DRB1*04− group:57.45 ± 84.36 μm, *p* = 0.807).

Regression analysis was performed with final visual acuity, age, sex, DRB1*04+/−, first CCT, first CRT, and first BCVA as objective variables. DRB1*04+/− and sex showed a significant difference (*p* = 0.0231 and *p* = 0.0226, respectively).

No significant difference was found for the other parameters (age: *p* = 0.90, first CRT: *p* = 0.17, first CCT: *p* = 0.14, first visual acuity: *p* = 0.17).

## Results in other HLA

We identified 5 patients as HLA- A * 24:02 the most common of the HLA- A (22.7%) .11 patients had DRB1*04,8 patients had A * 26,DRB*9 had 10 patients and only 2 people as B*51(Table 2).

HLA*A26 showed no significant difference in visual acuity and CCT after 6 months (VA: *p* = 0.140, CCT: *p* = 0.541). HLA B*9showed no significant difference in visual acuity and CCT after 6 months (VA: *p* = 0.515, CCT: *p* = 0.956). HLA*B51 was not analyzed due to its small number (only 2 patients).

There were no significant associations between the HLA alleles and VA, CCT, CRT except HLA-DR*4. Hardy-Weinberg equilibrium was not rejected for HLA-DR*4 (Χ^2^: 0.468).

### Availability of data and materials

The datasets used and/or analyzed during the current study are available from the corresponding author on reasonable request.

## Discussion

We identified a clinically interesting relationship between HLA and visual function in the unclassifiable uveitis group. In the present study, we have shown that that DRB1*04 may be related to CCT and post-treatment visual acuity in Japanese patients, excluding those with VKH disease. The relationship between VKH and HLA has long been known [5]. A report from Korea, also in Asia, reported an association between the risk of developing VKH and HLA [5]. Thus, it is considered that HLA, which is involved in immunity, is closely related to VKH. In our previous study investigating VKH disease and DRB1*04, we showed that post-treatment vision may be better with DRB1*04 [19].

Some possible reasons for the different results are as follows. The previous report examined only patients with VKH disease. In addition, all patients were treated with methylprednisolone. There were no cases of treatment with intravenous methylprednisolone in this study. The reason for the difference in the results between the previous study and the current study may be related to HLA response to treatment. In this study, we included unclassifiable uveitis, and the degree of symptoms differed and the treatment varied accordingly. CCT decreased in both the DRB1*04+ and DRB1*04− groups after treatment. We found no difference in the amount of change in CCT and no effect on vision.

Anatomically, the uvea is considered to be a blood flow-rich tissue with an active immune response. However, the blood-retinal barrier exists in the blood vessels of the eye, precluding a normal immune response. However, when uveitis occurs, the blood-retinal barrier is disrupted, which allows recognition of self-antigens in the presence of inflammation [5, 7].

HLA molecules are categorized into two classes: class I and class II. HLA-DRB1*04 is classified as MHC class II, which presents antigens to CD4+ T cells at the site of infection [23]. In other words, HLA is involved in the presentation of antigens to CD4+ T cells (Helper T cells), and HLA is likely involved in T cell-involved inflammation. In fact, VKH disease patients show an inflammatory response to melanin due to sensitization to melanocyte epitopes. In particular, patients with HLA-DRB1*0405 recognize a broader melanocyte-derived peptide repertoire [24]. The pathology of sympathetic ophthalmitis also shows an accumulation of T cells in the choroid [25]. T cells and HLA may also be involved in inflammation in the choroid. Activation of the local immune response is indicated by the upregulation of HLA class II molecules on the retinal pigment epithelium and choroidal melanocytes [26]. Furthermore, immunochemical examination of human eye samples of age-related macular degeneration showed the presence of the leukocyte antigens CD45, CD4, CD8, CD14, and CD83 in the choroid [27]. Based on these findings, the results of HLA genotyping may correlate with morphological changes in the choroid even in unclassifiable uveitis, excluding VKH disease. In our current results, the CCT was thicker in the HLA-DRB1*04+ group. The choroidal immune response associated with MHC interacts with T cells including CD4+ T cells. In this study, the CCT at baseline was thicker in the HLA-DRB1*04 group. This may be because HLA is involved in choroidal inflammation and the HLA-DRB1*04 group is more responsive to inflammation, because in previous article of autoimmunity area including rheumatoid arthritis, described that HLA-DRB1*04 group implicate those residues around the putative antigen binding site of the DR beta molecule in the pathogenesis. These data provide a basis for understanding host susceptibility to inflammatory diseasies at a molecular level [28, 29]. Choroidal blood vessels, including choriocapilalis, enhances autoimmune interstitial inflammation, secondary vasodilation occurs, and interacts with interstitial inflammatory edema to thicken the choroid on the OCT. It may be bringing.

No reports have described an association among various types of non-infectious uveitis, HLA, and vision. HLA-B27-positive acute anterior uveitis, worsening of visual acuity occurs in HLA-B27+ patients compared to HLA-B27− patients [17]. The report also cites worsening cataracts as one of the causes of worsening vision. However, HLA-B27 is a class I MHC molecule and may have a completely different mechanism than HLA-DRB1*04. The number of patients with HLA-B27 was small and could not be considered in this study. Therefore, the actual impact is unknown. In addition, we identified HLA-A*24:2 with the highest rate. This is consistent with the HLA distribution in the Japanese population [30]. However, the distribution was different, which may be due to the small number of cases.

The mechanism that directly explains the relationship between HLADR and visual outcomes is unclear. Reports show a relationship between the choroid and visual acuity in pathological myopia [31]. However, pathological myopia involves a structural abnormality of the retina. In an earlier report, not only the choroid but also retinal structures affected the visual prognosis. Patients with HLA-DRB1*04 have severe choroidal inflammation that may affect visual function as a result. The present study did not find an association between visual acuity and CCT, and a direct relationship between the choroid and visual function is unknown. In addition, no significant difference was observed in other class II HLA. Inflammation in uveitis activates interferon γ, which in turn induces HLA class II, which in turn induces an immune response by presenting antigens to CD4 of T cell [32]. The presence of DR4 causes a marked inflammatory response, and the inflammation may have caused an increase in choroidal blood flow and impaired blood flow [28, 29]. Inflammation and the resulting impaired blood flow may have increased the CCT. It is possible that the number of patients is small and the mechanism of action may be different, but if we can understand the effect of HLA other than HLA -DR4, we may be able to understand the relationship between molecular action and visual acuity and CCT.

Our results suggest the following: HLA may be useful for predicting post-treatment visual acuity. HLA and pretreatment visual acuity may be used to predict visual outcomes. Further studies may allow for the selection of treatment methods based on HLA.

This study has some important limitations, First, the study included only east Asian patients and was limited to the region in which the patients lived. Therefore, genetic polymorphisms may be limited. The number of patients analyzed in this study was small because it was limited to patients in whom HLA was assessed. In addition, the observation period was only 6 months, and the results may vary with longer observation. We hypothesized that HLA would affect reactivity and visual acuity in choroidal inflammation. However, there was no difference in visual acuity at baseline. The number of participants was also considered to be very small. We must consider fully that there is a selection bias. The results may change as the number of participants increases. Changes in CCT did not take into account age, gender, ocular axis, image registration. Past published reports have shown that CCT is affected by a variety of factors. Diurnal variation, axial length, and ethnic differences have also been shown to affect it [33–36]. No distinguishing between their correlation is the most problematic limitation of this paper. In addition, Our results may vary depending on the method of treatment. The main treatment was steroid eye drops, but the number and type of drops varied, and some patients received STTA. Therefore, the change may have been influenced by the intensity of treatment.

In conclusion, we have shown that the presence of HLA-DRB1*04 may affect post-treatment visual outcomes and CCT in unclassifiable uveitis patients.

## Supplementary Information

**Additional file 1.**
