# Supplementary material for: Visual outcomes and choroidal thickness associated with human leukocyte antigen DRB1*04 in unclassifiable uveitis in Japanese patients
Source: BMC Ophthalmol. 2021 Dec 28;21:457. doi: 10.1186/s12886-021-02222-9 (PMC8715637; doi:10.1186/s12886-021-02222-9)
Supplement: Supplementary file 1 — Additional file 1. [file 12886_2021_2222_MOESM1_ESM.docx]

Visual outcomes and choroidal thickness associated with human leukocyte antigen DRB1*04 in unclassifiable uveitis in Japanese patients

Norihiko Misawa^1^, Mizuki Tagami^1^, Atsushi Sakai^1^, Takeya Kohno^1^,

Shigeru Honda^1^

1. Department of Ophthalmology and Visual Sciences, Graduate School of Medicine, Osaka City University, Osaka, Japan

**Corresponding author:**

Mizuki Tagami

Department of Ophthalmology and Visual Sciences

Graduate School of Medicine, Osaka City University

1-5-7 Asahimachi, Abeno-ku, Osaka-shi, Osaka 545-8586, Japan

Tel: +81- 6-6645-3867, Fax: +81-6645-3867

E-mail: tagami.mizuki@med.osaka-cu.ac.jp

localID Sex sex age HLADR1 HLADR2 HLADQ1 HLADQ2 HLAA1 HLAA2 HLAB1 HLAB2 FirstLogMAR firstCRT firstCCT 6mLogMar 6MCRT 6MCCT

1 1 male 47 4 9 4 3 24 31 54 56 0.096910013 187 255 0.096910013 661 402

2 2 female 79 8 9 6 3 24 24 61 61 0 475 225 -0.079181246 276 112

3 1 male 72 4 9 4 9 2 26 46 48 0.15490196 270 477 0.301029996 362 258

4 2 female 67 15 9 6 9 2 24 61 46 0.096910013 412 327 -0.079181246 165 216

5 1 male 81 15 4 6 4 24 24 7 52 0.045757491 294 300 0.698970004 89 107

6 2 female 49 12 14 7 9 24 26 62 54 -0.176091259 175 147 -0.176091259 181 183

7 2 female 47 8 14 6 5 26 31 62 35 -0.079181246 221 184 -0.176091259 232 173

8 2 female 71 4 9 8 9 24 26 61 46 0.045757491 217 253 0 191 225

9 1 male 36 4 12 4 7 24 24 55 59 -0.176091259 256 375 -0.079181246 228 375

10 2 female 70 4 8 4 6 2 24 46 60 0.301029996 575 222 0 439 280

11 1 male 47 4 9 4 9 2 24 51 51 0.22184875 226 436 0.22184875 266 476

12 1 male 62 9 12 7 9 2 2 13 61 0.397940009 491 603 0.045757491 332 390

13 2 female 38 4 15 4 6 2 24 51 61 0 217 604 -0.079181246 236 330

14 1 male 76 9 9 9 9 26 26 61 61 -0.079181246 166 463 0 214 338

16 2 female 63 15 9 6 9 24 26 39 61 0.045757491 483 238 0 243 170

17 2 female 33 15 10 6 5 1 2 37 39 -0.079181246 203 183 -0.079181246 209 160

18 1 male 43 4 15 4 6 2 24 61 52 0.15490196 295 308 0.301029996 230 323

19 2 female 49 4 4 4 4 24 26 54 59 0.698970004 1212 410 0.301029996 182 367

20 1 male 26 8 14 8 5 24 24 35 61 0.301029996 215 206 0.301029996 663 380

21 2 female 45 1 15 5 6 24 24 7 52 -0.176091259 340 145 -0.079181246 276 132

22 2 female 25 4 14 4 5 2 26 35 55 0.698970004 439 336 1 158 225

10L 2 female 70 4 8 4 6 2 24 46 60 0.096910013 316 287 0 271 217

11L 1 male 47 4 9 4 9 2 24 51 51 -0.176091259 217 450 0.15490196 266 406

12L 1 male 62 9 12 7 9 2 2 13 61 -0.176091259 256 466 0 254 326

13L 2 female 38 4 15 4 6 2 24 51 61 -0.079181246 251 552 -0.176091259 236 320

14L 1 male 76 9 9 9 9 26 26 61 61 0 124 404 0 147 295

15L 1 male 47 1 9 5 9 24 24 27 52 0.522878745 243 264 -0.079181246 268 186

16L 2 female 63 15 9 6 9 24 26 39 61 0 537 240 0 230 194

17L 2 female 33 15 10 6 5 1 2 37 39 -0.176091259 213 140 -0.176091259 204 150

18L 1 male 43 4 15 4 6 2 24 61 52 0.698970004 256 346 0.045757491 256 343

19L 2 female 49 4 4 4 4 24 26 54 59 0.522878745 1066 607 0.397940009 211 302

1L 1 male 47 4 9 4 3 24 31 54 56 0.045757491 324 284 1 1517 135

21L 2 female 45 1 15 5 6 24 24 7 52 -0.176091259 289 201 -0.176091259 267 171

22L 2 female 25 4 14 4 5 2 26 35 55 -0.176091259 191 137 -0.079181246 184 186

2L 2 female 79 8 9 6 3 24 24 61 61 0.096910013 536 278 -0.079181246 275 118

3L 1 male 72 4 9 4 9 2 26 46 48 0 197 325 -0.079181246 198 247

4L 2 female 67 15 9 6 9 2 24 61 46 -0.079181246 166 164 -0.079181246 214 144

5L 1 male 81 15 4 6 4 24 24 7 52 0 292 276 0.698970004 86 63

6L 2 female 49 12 14 7 9 24 26 62 54 -0.176091259 183 205 -0.079181246 171 203

7L 2 female 47 8 14 6 5 26 31 62 35 -0.079181246 483 543 -0.176091259 217 436

8L 2 female 71 4 9 8 9 24 26 61 46 -0.176091259 203 230 -0.176091259 206 269

9L 1 male 36 4 12 4 7 24 24 55 59 -0.176091259 233 472 -0.079181246 273 436
